# First-principles study of coadsorption of Cu^2+^ and Cl^−^ ions on the Cu (110) surface

**DOI:** 10.1039/c9ra10072e

**Published:** 2020-02-26

**Authors:** Khoong Hong Khoo, Bharathi Madurai Srinivasan, Ramanarayan Hariharaputran, Chaitanya Amol Joshi, David Wu Tai-Yen, Hongmei Jin

**Affiliations:** Institute of High Performance Computing, Agency for Science, Technology and Research (A*STAR) 1 Fusionopolis Way, Connexis Singapore 138632 Singapore jinhm@ihpc.a-star.edu.sg

## Abstract

Motivated by the importance of Cl^−^ in the industrial electrolytic Cu plating process, we study the coadsorption of Cl^−^ and Cu^2+^ on the Cu (110) surface using first-principles density functional theory (DFT) calculations. We treat the solvent implicitly by solving the linearized Poisson–Boltzmann equation and evaluate the electrochemical potential and energetics of ions with the computational hydrogen electrode approach. We find that Cl^−^ alone is hardly adsorbed at sufficiently negative electrochemical potentials *μ*_Cl_ but stable phases with half and full Cl^−^ coverage was observed as *μ*_Cl_ is made more positive. For Cl^−^ and Cu^2+^ coadsorption, we identified five stable phases for electrode biases between −2*V* < *U*_SHE_ < 2*V*, with two being Cl^−^ adsorption phases, two being Cl^−^ + Cu^2+^ coadsorption phases and one being a pure Cu^2+^ adsorption phase. In general, the free energy of adsorption for the most stable phases at larger |*U*_SHE_| are dominated by the energy required to move electrons between the system and the Fermi level of the electrode, while that at smaller |*U*_SHE_| are largely dictated by the binding strength between Cl^−^ and Cu^2+^ adsorbates on the Cu (110) substrate. In addition, by studying the free energy of adsorption of Cu^2+^ onto pristine and Cl^−^ covered Cu (110), we conclude that the introduction of Cl^−^ ion does not improve the energetics of Cu^2+^ adsorption onto Cu (110).

## Abstract 2

Free energy of adsorption for the most stable phases predicted by DFT calculations as a function of electrode potential.

## Graphical abstract

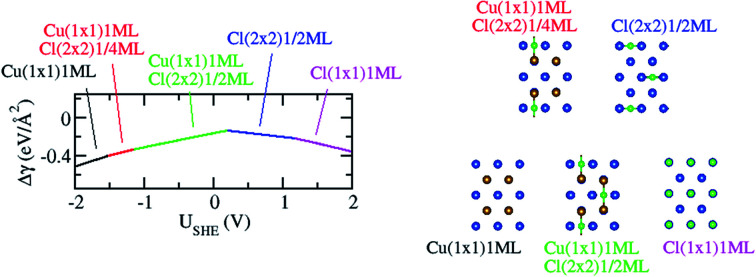

## Introduction

Copper is a ubiquitous metal of great importance due to its excellent physical properties such as mechanical strength, chemical stability as well as high electrical and thermal conductivities. This allows copper to be utilized in numerous applications such as catalysis, heat exchange and electrical wiring. In particular, copper is used extensively as interconnects in modern electronic circuits where the copper metallization is achieved through electrodeposition.^1^ In this process, plating additives are typically introduced to ensure that the plating of features such as vias and trenches are complete, as preferential plating at the opening might lead to pinch off and leave a void in the interconnect.^2^ To avoid this scenario, a plating suppressor is added that resides on the feature surface to inhibit plating, while an accelerator is added that is present mostly at the feature bottom to increase plating rates.^2–9^ This promotes ‘bottom-up’ filling and is the key to prevent void formation. Apart from the suppressor and accelerator, the next most important additive is the chloride ion (Cl^−^), as it is one of the most common constituents of commercial acid-copper plating baths. A small amount of Cl^−^ is essential as it affects the surface appearance, structure, and crystallographic orientation of the deposits, and it is also known to exert a synergetic effect with additives such as bis(3-sulfopropyl) disulfide [SPS] and polyethylene glycol [PEG].^10–15^ In the past, extensive experimental and theoretical studies were carried out to uncover the working mechanism of PEG and SPS in the presence of Cl^−^.^16,17^ There also exists a variety of experimental and theoretical studies^18–29^ for Cl^−^ adsorption on copper due to other industry applications; but less work was reported on the understanding of the Cl^−^ effect under copper electroplating environments. In view of the importance of Cl^−^ addition in the industrial plating process, a detailed study of chloride adsorption, especially ones that consider solvent and applied electrode potential effects, is necessary as it will serve as a good foundation for further studies into more complex situations including plating with multiple additives. Therefore, in this research, we perform density functional calculations to investigate the adsorption behaviour of Cl^−^ and Cu^2+^ on Cu(110) surface. We model solvent effects by solving the linearized Poisson–Boltzmann equation, and tune the electrode potential by adding or removing electrons to the simulation system. In this way, the energetics of Cl^−^ adsorption and Cl^−^ and Cu^2+^ coadsorption on Cu(110) surface are evaluated. We choose Cu(110) as this surface is commonly observed during acid-copper plating but was less commonly investigated in previous literature.

## Methodology

From the thermodynamic point of view, the most stable adsorption configuration can be predicted by identifying the structure that minimizes the Gibbs free energy of adsorption per unit surface area1
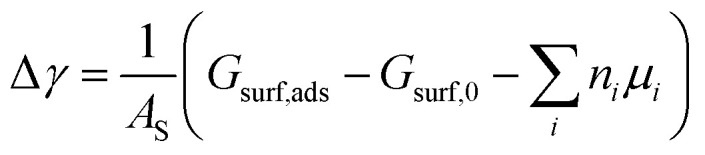
Here, *G*_surf,ads_ and *G*_surf,0_ are the Gibbs free energies of the ion adsorbed and pristine surfaces, *n*_i_ is the number of *i*th ion adsorbed, *μ*_i_ is the electrochemical potential of the ith ion in solution, and *A*_S_ is the surface area of the calculation cell.^28^ Since the terms TS and pV are rather small for solids, it is reasonable to neglect these dependencies and equate *G*_surf,ads_ and *G*_surf,0_ to the total energies obtained from DFT calculations, *i.e. G*_surf,ads_ = *E*_surf,ads_ and *G*_surf,0_ = *E*_surf,0_. To compute the electrochemical potentials of the ions *μ*_i_, we use the concept of the computational hydrogen electrode generalized to arbitrary ions.^29^ Under standard conditions, the free energy of a singly charged positive ion plus that of an electron at the Fermi level differs from the free energy of its bulk phase by *eU*^0^, where *U*^0^ is the standard reduction potential. This relation allows us to obtain *μ*_i_ without having to calculate the solvation energy of ions, but instead derive it from the bulk energy of the material in question. To obtain *μ*_i_ under general thermodynamic conditions, we just correct for the electrode potential with a *eU*_SHE_ term and the ion concentration with a *k*_B_*T* ln(*a*) term, where *U*_SHE_ is the electrode potential relative to the standard hydrogen electrode and *a* is the ion activity. Thus, we have2
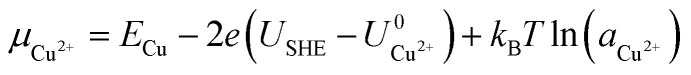
3
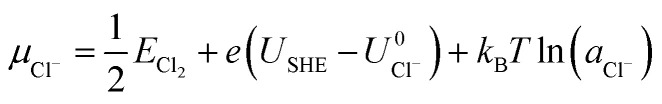
where *E*_Cu_ and *E*_Cl_2__ are the energies of a Cu atom in bulk Cu and Cl_2_ molecule respectively, both of which are obtained from DFT calculations. 
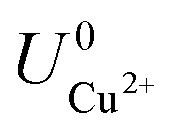
 and 
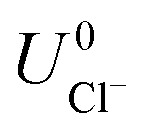
 are the standard reduction potentials and *a*_Cu^2+^_ and *a*_Cl^−^_ are the activities of the Cu^2+^ and Cl^−^ ions respectively. We can also rewrite eqn (1) in a different way by regrouping the terms,

4 Δ*γ* = (*E*_ads_ − *n*_Cl_Δ*μ*_Cl^−^_ − *n*_Cu_Δ*μ*_Cu^2+^_)/*A*_S_

where

5 *E*_ads_ = *E*_surf,ads_ − *E*_surf,0_ − *n*_Cl_*E*_Cl_2__/2 − *n*_Cu_*E*_Cu_

6
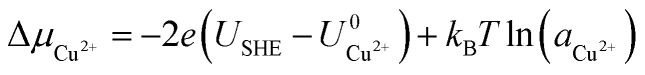
7
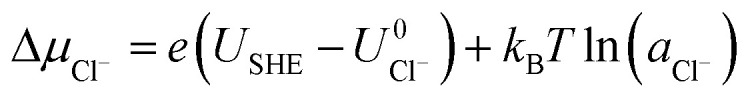


This way, we separate quantities obtained by first-principles calculations from those that are defined by external conditions.

To compute the total energies *E*_surf,ads_ and *E*_surf,0_, we perform DFT calculations on atomic slabs with 40.0 Å of vacuum perpendicular to the surface normal. Adsorbates are introduced on both sides of the surface to ensure that the interface dipole is symmetric on both faces. In addition, the electrode potential can be tuned in our simulation by adding or removing electrons to the system, as this becomes surface charge that creates an electric field near the slab which changes the work function. The corresponding electrode bias is then *U*_SHE_ = *W* − 4.44*V*, where 4.44*V* is the standard potential of the hydrogen electrode and *W* is the work function of the system. We also model the electrolyte solution with an implicit solvent method.^30,31^ Here, we solve the linearized Poisson–Boltzmann equation in place of the standard Poisson equation to account for screening effects of the ions and solvent. This effectively imposes a dielectric constant that varies from 1.0 in the solute to the bulk value of water in the vacuum region of the calculation. Also, there is an ionic charge term that varies linearly with potential and is parametrized by the Debye length. This addition enables the electric double layer to be represented more realistically and balances any excess surface charge.

Our calculations have been performed using the VASP package^32^ and the exchange correlation is described using the GGA-PBE density functional.^33^ Wavefunctions have been expanded using a plane wave basis with an energy cutoff of 500 eV and dispersion interactions are included using the DFT-D2 scheme.^34^ The ionic potentials are represented using the PAW formalism^35^ and the Brillouin zone is sampled with the spacing between *k*-points set to 0.3 Å^−1^. Geometry was optimized for the neutral system and convergence is reached when each force component is less than 0.01 eV Å^−1^. Electronic self-consistency is reached when the total energy is converged to within 10^−6^ eV. The implicit solvent dielectric permittivity is set to the bulk water value of 78.4 and the Debye screening length is determined by the ionic concentration and calculated using eqn (15) of ref. 31.

## Results and discussion

**Fig. 1:**
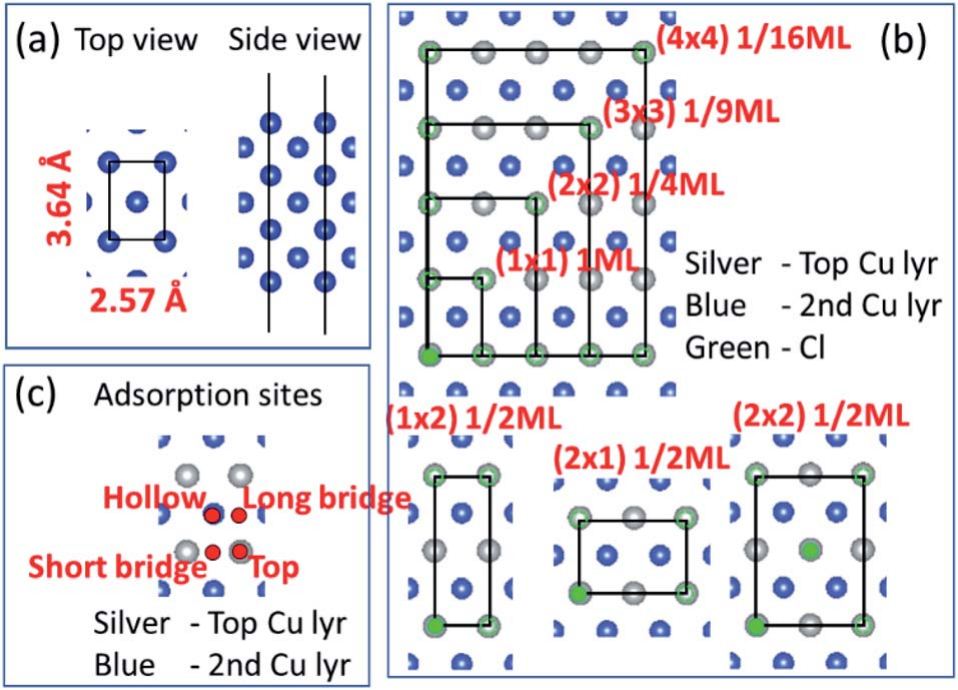
(a) Top and side views of the Cu(110) atomistic slab employed in our DFT calculations. (b) Top view of slab illustrating various two-dimensional supercells. Silver and blue atoms represent top and second Cu layers while green represents Cl atoms. (b) Only shows Cl on the top site but calculations also performed for hollow, long bridge and short bridge sites. (c) Hollow, long bridge, short bridge and top adsorption sites illustrated by red dots.

Before we consider Cu^2+^ and Cl^−^ coadsorption on the Cu (110) surface, let us first look at the simpler case of Cl^−^ adsorption. The starting point of our calculation is the Cu (110) surface, which we construct from a slab of 7 atomic layers thick as shown in Fig. 1a. The slab is oriented such that the (11̄0) and (001) directions are oriented along the *x* and *y* axes respectively. The in-plane lattice constants are obtained from DFT calculations on bulk Cu to give 2.57 Å and 3.64 Å for the (1 × 1) surface unit cell. We consider multiple adsorption configurations and adsorption sites for Cl on Cu (110) as shown in Fig. 1b. The structures considered for our calculations are denoted by (1 × 1) 1ML, (1 × 2) 1/2ML, (2 × 1) 1/2ML, (2 × 2) 1/2ML, (2 × 2) 1/4ML, (3 × 3) 1/9ML and (4 × 4) 1/16ML. For each of these structures, we consider Cl adsorbed on the top, long bridge, short bridge and hollow sites, as illustrated in Fig. 1c. We employ neutral slabs in these calculations and solvent effects are included using the implicit model described previously.

**Fig. 2:**
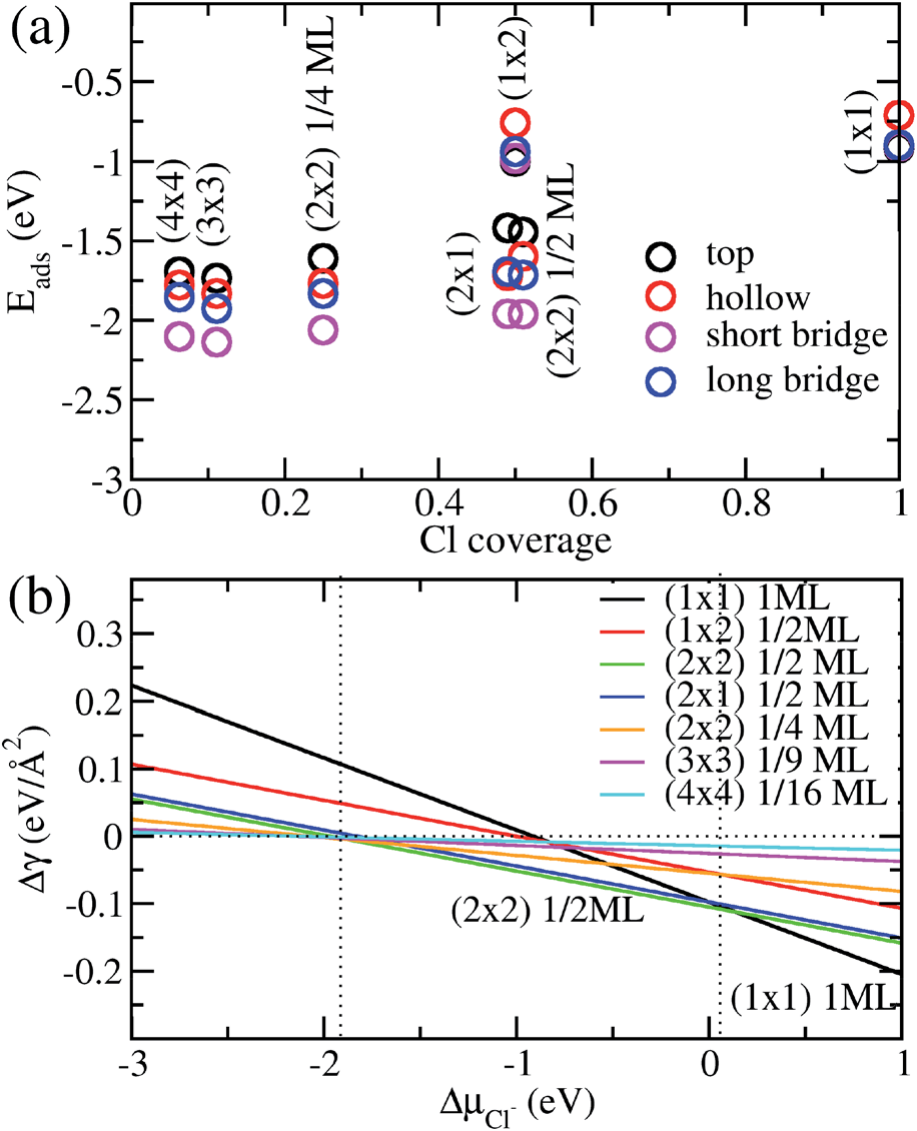
(a) Calculated adsorption energy *E*_ads_ per Cl^−^ ion on Cu(110) as a function of Cl^−^ coverage for different adsorption sites and configurations. (b) Free energy of adsorption Δ*γ* calculated as a function of the electrochemical potential Δ*μ*_Cl_ using eqn (4) and *E*_ads_ obtained above.

We show in Fig. 2a the adsorption energies *E*_ads_ of Cl on Cu (110). For a given type of adsorption site *i.e.* top, hollow, long or short bridge, the magnitude of *E*_ads_ decreases with increasing coverage due to electrostatic repulsion between negatively charged Cl ions.^23^ At low coverage, the repulsion is weaker due to larger distances between Cl adsorbates and screening by the Cu metal, as a result |*E*_ads_| decreases slowly. As the coverage increases, the repulsion energy becomes dominant and this can be seen in the dramatic decrease of |*E*_ads_| from 1/2ML to full coverage. At half coverage, we also see stark differences in the adsorption energy between the (1 × 2) 1/2ML, (2 × 1) 1/2ML, (2 × 2) 1/2ML configurations, which we similarly attribute to repulsion between Cl adsorbates. The adsorption energy |*E*_ads_| is weakest for the (1 × 2) 1/2ML system while that of (2 × 2) 1/2ML and (2 × 1) 1/2ML configurations are close to each other. This is because the nearest neighbour Cl–Cl distance for the (1 × 2) 1/2ML system is 2.57 Å, which leads to significantly larger electrostatic repulsion than in the (2 × 1) 1/2ML and (2 × 2) 1/2ML configurations, which have the nearest Cl–Cl distances of 3.64 Å and 4.46 Å respectively.

Another trend that is clear from Fig. 2a is that the short bridge adsorption site is consistently preferred over other sites except for the highest energy configurations (1 × 2) 1/2ML and (1 × 1) 1ML, where the top site is preferred. We believe the preference for the short bridge site is to maximize the binding between Cl and the Cu (110) substrate, as it represents the optimal balance between Cu–Cl distance and number of nearest neighbour Cu–Cl bonds. For example for the (2 × 1) 1/2ML structure, Cl in the short bridge site has two nearest neighbour Cu atoms that are 2.24 Å away, while the top configuration has one nearest neighbour Cu at a distance of 2.11 Å and the hollow site has four nearest neighbour Cu atoms with a Cu–Cl bond length of 2.64 Å. Conversely, for the high coverage configurations (1 × 2) 1/2ML and (1 × 1) 1ML where electrostatic repulsion dominates, the top adsorption site is preferred. This is because the amount of charge transferred is lower owing to reduced interaction between Cl and the Cu substrate. This reduction in electrostatic repulsion more than offsets the lowering in Cu–Cl binding to make the top site the energetically preferred site. In particular, Bader charge analysis shows that the charge transfer to each Cl atom in the (1 × 1) 1ML structure is 0.30*e* for the short bridge and hollow sites, 0.27*e* for the long bridge site and 0.24*e* for the top site. Thus we see that the magnitude of adsorption energy increases with decreasing charge transfer to Cl.

Now that we have determined the energetically preferred Cl adsorption sites for the various configurations, we compute the free energy of adsorption Δ*γ* using eqn (4) for each of these configurations. Specifically, we compute Δ*γ* for the (2 × 1) 1/2ML, (2 × 2) 1/2ML, (2 × 2) 1/4ML, (3 × 3) 1/9ML, (4 × 4) 1/16ML structures with Cl adsorbed on the short bridge site and (1 × 1) 1ML, (1 × 2) 1/2ML configurations with Cl adsorbed on the top site. The effects of the applied electrode bias and ion activity are represented through the electrochemical potential Δ*μ*_Cl^−^_ and *E*_ads_ is obtained from our previous neutral slab calculations. Also, since the slope of Δ*γ vs.* Δ*μ*_Cl^−^_ is only dependent on the coverage, we only need to consider the lowest adsorption energy site for a given coverage to identify the state with the lowest Δ*γ*. Using this procedure, we generate a plot of Δ*γ* as shown in Fig. 2b, where we identify three stable phases over the range of Δ*μ*_Cl^−^_. If we focus on the region near Δ*μ*_Cl^−^_ ∼ 0 eV, we have Δ*γ* = *E*_ads_/*A*_S_ and we find that the (1 × 1) 1ML, (2 × 2) 1/2ML and (2 × 1) 1/2ML configurations almost equally stable with the lowest free energies. However as one increases the electrochemical potential Δ*μ*_Cl^−^_, the (1 × 1) configuration becomes most favourable as the slope −*n*_*i*_/*A*_S_, which is the negative of the areal density of Cl adsorbates, is most negative for the (1 × 1) system. For the intermediate region below Δ*μ*_Cl^−^_ ∼ 0 eV, the potential term −*n*_*i*_Δ*μ*_Cl_/*A*_S_ in eqn (4) now acts to increase Δ*γ* faster for the (1 × 1) 1ML configuration, and the (2 × 2) 1/2ML and (2 × 1) 1/2ML structures are now the most favourable. This is in agreement with previous LEED work that observed a 0.5 coverage *c*(2 × 2) Cl adsorption on Cu (110) exposed to HCl.^26^ However as we keep decreasing the electrochemical potential Δ*μ*_Cl^−^_ to the point beyond ∼−1.9 eV, the potential term becomes large enough to dominate over the adsorption energy and Δ*γ* becomes positive for all the systems considered, hence no adsorption occurs. This is because the energy required to deposit an electron from the ion into the electrode becomes energetically too costly.

In the next step, we look at the coadsorption of Cu^2+^ and Cl^−^ ions on a Cu (110) surface under different electrode potentials and ion concentrations. In these calculations, we consider Cl^−^ adsorption configurations studied above but limit the coverage to the range 1/4 to 1, as lower coverages were found to be energetically unfavourable. Also, we employ the optimal Cl^−^ adsorption sites found in the preceding section in our coadsorption study. This leads to the calculation geometries of (1 × 1) 1ML and (1 × 2) 1/2ML on the top site and (2 × 1) 1/2ML, (2 × 2) 1/2ML and (2 × 2) 1/4ML configurations on the short bridge site. For Cu^2+^ adsorption, we consider the same configurations as Cl^−^ adsorption, *i.e.* (1 × 1) 1ML, (1 × 2) 1/2ML, (2 × 1) 1/2ML, (2 × 2) 1/2ML and (2 × 2) 1/4ML. However, we assume that adsorption occurs on the hollow site, as this is the position that leads to the formation of bulk Cu. The possible configurations for Cu^2+^ and Cl^−^ adsorption are summarized in Table 1, and we take all possible combinations of the Cu^2+^ and Cl^−^ configurations to generate 36 co-adsorption systems.

**Table 1:** Respective adsorption sites for Cl^−^ and Cu^2+^ ions on Cu(110) considered in our co-adsorption study

Cl^−^ adsorption sites	Cu^2+^ adsorption sites
No adsorption	No adsorption
(1 × 1) 1ML top	(1 × 1) 1ML hollow
(1 × 2) 1/2ML top	(1 × 2) 1/2ML hollow
(2 × 1) 1/2ML short bridge	(2 × 1) 1/2ML hollow
(2 × 2) 1/2ML short bridge	(2 × 2) 1/2ML hollow
(2 × 2) 1/4ML short bridge	(2 × 2) 1/4ML hollow

We again apply eqn (4) to compute Δ*γ* for coadsorption, however we introduce between −0.4, −0.2, 0.0, 0.2, 0.4 electrons to the Cu (110) slabs under consideration to obtain *E*_surf,ads_ and *E*_surf,0_ for different applied potentials *U*_SHE_. The potential is evaluated by computing the work function *W* under different charging conditions and applying the relation *U*_SHE_ = *W* − 4.44*V*, giving rise to a potential variation on the order of 1*V*. The resulting total energies are fit to a quadratic polynomial in *U*_SHE_ so that the total energy can be evaluated at arbitrary *U*_SHE_. In general, we set

8 *E*_surf,ads_ = *aU*_SHE_^2^ + *bU*_SHE_ + *c*

9 *E*_surf,0_ = *a*_0_*U*_SHE_^2^ + *b*_0_*U*_SHE_ + *c*_0_

then eqn (4)–(6) can be combined with eqn (8) and (9) to give10
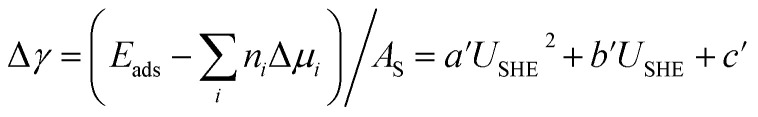
where *a*′ = (*a* − *a*_0_)/*A*_S_

11 *b*′ = (*b* − *b*_0_ − *n*_Cl_*e* + 2*n*_Cu_*e*)/*A*_S_

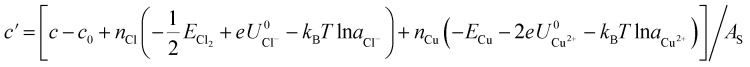


**Fig. 3:**
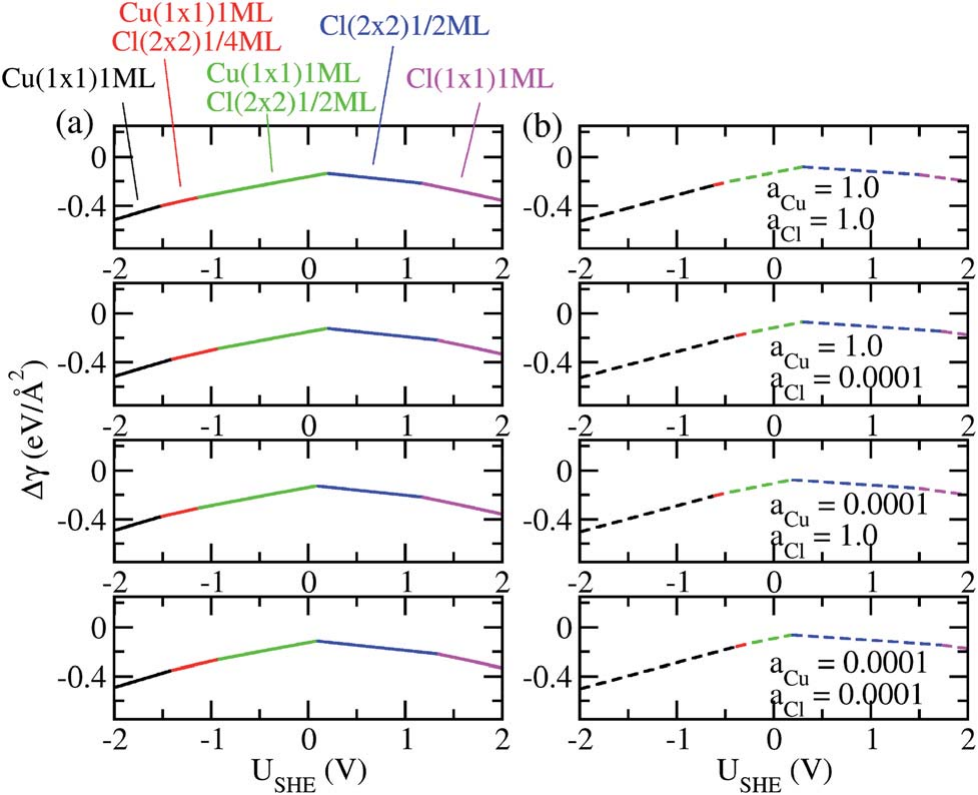
(a) Plot of free energy of adsorption Δ*γ* for most stable phases as function of electrode potential *U*_SHE_, with *E*_surf,ads_ and *E*_surf,0_ obtained using charged calculations. (b) Same as (a) except *E*_surf,ads_ and *E*_surf,0_ are obtained from neutral cell calculations.

The coefficients *a*, *b*, *c* as well as *a*_0_, *b*_0_ and *c*_0_ are obtained from fits using total energies obtained from charged DFT calculations, and *E*_Cu_ and *E*_Cl_2__ are obtained from separate calculations on bulk fcc Cu and an isolated Cl_2_ molecule respectively. The coefficients *a*′, *b*′ and *c*′ are computed for each of the 36 co-adsorption configurations and the Δ*γ* are compared at each *U*_SHE_ to identify the phase that minimizes Δ*γ*. This procedure is repeated for the activity combinations (*a*_Cl^−^_ = 1, *a*_Cu^2+^_ = 1), (*a*_Cl^−^_ = 1, *a*_Cu^2+^_ = 0.0001), (*a*_Cl^−^_ = 0.0001, *a*_Cu^2+^_ = 1) and (*a*_Cl^−^_ = 0.0001, *a*_Cu^2+^_ = 0.0001) and the results are plotted in Fig. 3a.

As can be seen, there are five preferred phases over a range of *U*_SHE_ between −2.0 to 2.0 V. Inspection of the plots shows that the nature of the stable configurations and shape of the Δ*γ* plots are mostly independent of the activities *a*_Cl_ and *a*_Cu_. This is because the term *k*_B_*T* ln(*a*) is much smaller than other energy terms in Δ*γ* at room temperature *k*_B_*T* = 0.025 eV in the studied activity range. We conclude that the activity term only matters if the ion is present in very small concentrations. We list the *a*′, *b*′ and *c*′ values of the 5 stable phases in Table 2. For all the 36 phases considered, we have found that the values of *a*′ ranges between −0.020 to 0.028 eV Å^−2^, that of *b*′ ranges between −0.276 to 0.436*e* Å^−2^, and *c*′ is between −0.326 and −0.001 eV Å^−2^. Comparing the possible values of *a*′ and *b*′, it can be concluded that the quadratic term is generally smaller than the linear term for the range of *U*_SHE_ considered and is therefore not consequential in determining the most stable phase. Near the extremities of the *U*_SHE_ range considered, the most stable phases Cu (1 × 1) 1ML and Cl (1 × 1) 1ML correspond to the systems with the largest magnitudes of *b*’ for the systems considered, and these large values originate from the large areal densities *n*/*A*_S_ arising from the full coverage of adsorbates. Conversely for small values of |*U*_SHE_|, both quadratic and linear terms fall away and Δ*γ* is dominated by the constant *c*′ term. The contribution of this term is mostly the adsorption energy *E*_ads_ plus the reduction potential term 
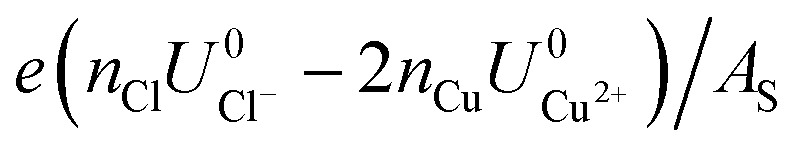
, with 
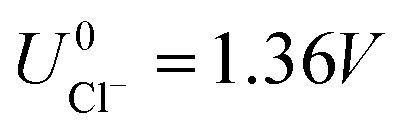
 and 
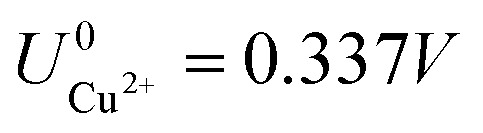
. This implies that stable configurations are the ones with low *E*_ads_ along with the ones with large *n*_Cu_ due to the 
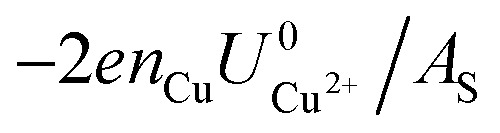
 term, which gives rise to the Cl^−^ (2 × 2) 1/2ML configuration owing to its low *E*_ads_ and Cu (1 × 1) 1ML due to its large *n*_Cu_.

**Table 2:** Coefficients of Δ*γ* quadratic fit for the stable coadsorption phases identified for the range |*U*_SHE_| < 2V

	*a*′ (eV Å^−2^)	*b*′ (e Å^−2^)	*c*′ (eV Å^−2^)
Cu (1 × 1) 1ML	0.01085	0.436	−0.117
Cu (1 × 1) 1ML Cl (2 × 2) 1/4ML	0.00347	0.375	−0.244
Cu (1 × 1) 1ML Cl (2 × 2) 1/2ML	−0.00981	0.289	−0.326
Cl (2 × 2) 1/2ML	−0.00236	−0.164	−0.237
Cl (1 × 1) 1ML	−0.0200	−0.276	−0.0831

As a test of the charging formalism used in this study, we also compute the total energies *E*_surf,ads_ and *E*_surf,0_ using neutral slabs for the 36 possible co-adsorption configurations, and generated a *U*_SHE_ independent *E*_ads_ for computing Δ*γ*. The results of these calculations are seen in Fig. 3b, where we see the same five phases emerging as the most stable, with the shape of the Δ*γ* curve remaining unchanged from the charged calculations. However, the boundaries separating each phase has shifted, and this is particularly pronounced for the boundary between Cu (1 × 1) 1ML and Cu (1 × 1) 1ML + Cl (2 × 2) 1/4ML. From this, it seems that charging the slabs has stabilized Cl^−^ adsorption in the presence of Cu^2+^ relative to neutral calculations, however the main conclusions between charged and uncharged calculations remain unchanged and there is semi-quantitative agreement between results from these two sets of data.

**Fig. 4:**
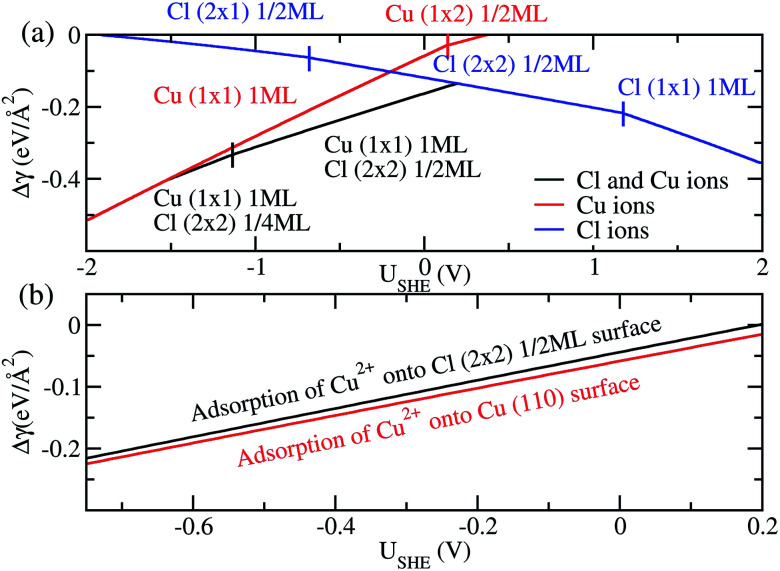
(a) Free energy of adsorption Δ*γ* for the most stable phases on Cu (110) in the presence of Cl^−^ ions (blue), Cu^2+^ ions (red) and both Cu^2+^ and Cl^−^ ions (black). (b) Free energy of adsorption Δ*γ* for Cu^2+^ on pristine Cu (110) surface (red) and Cl (2 × 2) 1/2ML surface (black) to form a Cu (1 × 1) 1ML adlayer.

Finally, we compare the adsorption of only Cl^−^ or Cu^2+^ ions *vs.* coadsorption of both Cu^2+^ and Cl^−^ on Cu (110). To do this, we have calculated the Δ*γ* for the most stable configurations in the presence of Cl^−^ ions only, Cu^2+^ ions only and both Cl^−^ and Cu^2+^ ions and plotted them in Fig. 4a, with the activities *a*_Cl^−^_ and *a*_Cu^2+^_ kept at 1.0.

For regions where |*U*_SHE_| is large, the most stable phases consist of adsorption of only a single type of ion. However, in the intermediate region of *U*_SHE_ between −1.4*V* and 0.2*V*, the coadsorption of both Cu and Cl ions is energetically preferable. In addition, we can determine if the presence of Cl^−^ promotes the adsorption of Cu^2+^ on Cu (110). To do this we consider the following reactions Cl (2 × 2) 1/2ML + Cu^2+^ → Cu (1 × 1) 1ML + Cl (2 × 2) 1/2ML and Cu(110) + Cu^2+^ → Cu (1 × 1) 1ML. The first reaction is for Cu^2+^ adsorption on a Cl^−^ covered Cu surface and the second is for Cu^2+^ on pristine Cu, and we expect these states to be stable in the potential range −0.68*V* < *U*_SHE_ < 0.15*V*. The free energy of adsorption for these reactions can be obtained by taking the difference between the black and blue curves of Fig. 4a for the former and the red curve for the latter. As can be seen in Fig. 4b, Δ*γ* is almost identical for adsorption onto either the Cl adsorbed or pristine Cu (110). From this, we can see that the energetics of copper plating is fairly independent of the presence of Cl^−^ ions on the Cu surface, and this strongly suggests that the presence of Cl^−^ neither enhance nor deter the plating of Cu.

## Conclusions

In this work, we have studied the adsorption of Cl^−^ as well as coadsorption of Cu^2+^ and Cl^−^ on the Cu (110) surface using first-principles DFT calculations. The screening arising from the ionic solvent is modeled by solving the linearized Poisson–Boltzmann equation, and the electrode potential is tuned by adding or removing charge to the simulation system. By calculating the free energy of adsorption, we identify the preferred phases of Cl^−^ on Cu(110) as the Cl (1 × 1) 1ML for positive Δ*μ*_Cl^−^_ and Cl (2 × 2) 1/2ML for negative Δ*μ*_Cl^−^_, while for Δ*μ*_Cl^−^_ < −1.9 V, there are no stable adsorbed phases of Cl^−^. In addition, we find that for Cl^−^ configurations at low coverage, the short bridge adsorption site is preferred as it maximizes binding between the Cl^−^ adsorbate and Cu substrate, whereas for high coverage configurations where electrostatic repulsion between Cl^−^ is dominant, the top configuration is more favorable as it minimizes charge transfer from Cu to Cl^−^. For our study of Cu^2+^ and Cl^−^ coadsorption on Cu (110), we identify five different stable phases over a range of *U*_SHE_, with the configurations at large |*U*_SHE_| dominated by full coverage of either Cl^−^ or Cu^2+^ adsorbates while that of low |*U*_SHE_| being structures that have large adsorption energies *E*_ads_, such as Cl (2 × 2) 1/2ML. In addition, we found that calculating Δ*γ* using neutral slab calculations gives semi-quantitative agreement with charged slab calculations that explicitly model the electrode potential, and that the presence of Cl^−^ does not significantly enhance or deter the plating of Cu^2+^.

## Conflicts of interest

There are no conflicts to declare.

## Supplementary Material
